# Multi-criterial evaluation of P-removal optimization in rural wastewater treatment plants for a sub-catchment of the Baltic Sea

**DOI:** 10.1007/s13280-017-0977-8

**Published:** 2017-11-21

**Authors:** Michael Cramer, Tatyana Koegst, Jens Traenckner

**Affiliations:** Institute for Water Management, University of Rostock, Satower Str. 48, 18059 Rostock, Germany

**Keywords:** Activated sludge model ASM, Cost-efficient measures, Multi-criterial prioritization, Phosphorous removal, Rural wastewater treatment plant, Water quality management

## Abstract

Rural wastewater treatment plants (WWTP) are recognized as a significant phosphorous (P)-emission source in the German sub-catchments of the Baltic Sea. But enhancement of P-removal is cost intensive especially for small communities. This study proposes a concept to efficiently reduce P-emissions from WWTP by introducing low-cost non-constructional measures and assessing the resulting change of ambient water quality on a river basin scale. As case study, we choose the Warnow catchment in Mecklenburg-Vorpommern (Germany) where 76 % of WWTP-emissions originate from treatment plants without P-discharge limits. For these facilities, we evaluated two modification options: (1) optimization of enhanced biological Phosphorous removal (EBPR) in activated sludge systems (ASS) and/or (2) chemical precipitation (where EBPR is applicable). The total operational optimization potential in the Warnow catchment is about 20 %. Further improvements can only be applied by a chemical precipitation, with the drawback of increasing the wastewater disposal cost up to 6.5 euros CAP^−1^a^−1^. To prioritize relevant plants for improved P-removal we proposed two evaluation criteria: combining absolute emission, and impact on ambient water quality and costs.

**Electronic supplementary material:**

The online version of this article (10.1007/s13280-017-0977-8) contains supplementary material, which is available to authorized users.

## Introduction

A main driver of eutrophication in ambient water systems is the load of Phosphorus (P)-fractions (Carpenter et al. 1998; Correll 1998; Vohla et al. 2011). Its minimization demands an efficient source control in the catchment. Independent from the actual sources partitioning in the system, point sources can be more easily targeted for control and management compared with diffuse sources scattered through a watershed. Therefore, the removal of P-load from municipal wastewater treatment plants (WWTP) is an essential and effective emission control strategy.

Generally, in the European Union discharge consents for P are only defined for large WWTP’s with more than 10^4^ population equivalents (PE). Accordingly, in Germany only WWTP’s larger than 10^4^ PE have to fulfil P-discharge limits. However, in rural areas, most of the WWTP are smaller than 10^4^ PE. For example, in the federal state of Mecklenburg-Vorpommern (MV, Germany) WWTP contribute with 18 % to total P-emission (Wendland et al. 2015). Based on the official survey data of MV, 61 % of this P-emission (63 of 103 t/a) can be assigned to WWTP with a capacity of less than 10^4^ PE (Table 1) (Tränckner et al. 2016). These data underline the need for P control also for small WWTP although so far no legal binding discharge limits exist.

**Table 1:** Parameter estimation for activated sludge systems in urban wastewater

	Size class 1	Size class 2	Size class 3	Size class 4	Size class 5
PE	< 1000	1001–5000	5001–10 000	10 001–100 000	> 100 000
P consent	None	None	None	2 mg_P_/L	1 mg_P_/L
Emitted P-load	27.3 (t/a)	25.2 (t/a)	10.1 (t/a)	29.8 (t/a)	10.3 (t/a)
Percentage	27	24	10	29	10

Typically, small WWTP’s are situated in rural areas with long specific sewer length, pressure systems, etc. making wastewater disposal often more cost intensive than in more densely populated regions. Therefore, costly upgrade measures to fulfil enhanced emission standards require sound cost-efficiency analyses. Mostly, operational measures are more cost-efficient than structural ones (Tränckner et al. 2016). Among other possibilities for the optimization of P-removal in a small WWTP (Hernandez et al. 2006; Vohla et al. 2011), the reduction of sludge retention time (SRT) (Merzouki et al. 2001), introduction of enhanced biological P-removal (EBPR) (López-Vázquez et al. 2008) and chemical precipitation (Fytianos et al. 1998) are the most promising technological solutions.

The SRT-reduction approach is based on the effect of producing more sludge by reducing decay in the activated sludge systems (ASS) (Henze 1987; DWA-A 131 2016). Whereas EBPR increases the P-content of the sludge by promoting the growth of P-storing bacteria (Smolders et al. 1995). This a sequence of anaerobic and aerobic conditions in AST, as prerequisite for the development of P-accumulating microorganisms (Tsuneda et al. 2006). In most cases, the increased sludge disposal costs, related to reduced SRT and introduction of EBPR, are compensated by the energy savings gained from reduced oxygen demand (Tränckner et al. 2016).

Where the optimization of biological P-removal is not sufficient or not applicable (e.g. near natural pond systems), P-removal can improved by chemical precipitation using Iron or Alum salts (Morse et al. 1998). This compels the installation of a dosage system, consumes the precipitants and produces additional sludge. For very small plants with low consumption of precipitants, compact dosage stations, consisting of a dosage pump directly fixed on a vessel, can be applied. For larger plants, container solutions where the precipitant is stored in an IBC container are a pragmatic solution.

Besides plant size, various structural and operational conditions as well as the required environmental effect are making the choice of the most suitable technology of P-removal or their combination with an individual task.

Since the majority of MV’s territory drains into the Baltic Sea, the reduction of P-emissions from rural WWTP is not only relevant for the receiving waters but also for this sensitive marine ecosystem. The catchment of the Warnow River has been chosen for the study as representative for the southern Baltic region. Within the total catchment area of 3250 km^2^ around 70 WWTP of 1–3 capacity size (i.e. without P-discharge limits) have been identified and assessed for their optimization potential with regard to P-removal. Since not all WWTP’s can be handled at once, a prioritization is needed.

Herewith, the main objective of this investigation is to evaluate the potential improvement of P-removal of the small WWTP in Warnow catchment on the low-cost operative basis and to assess the related effect on ambient water quality in the receiving water. For this, the following steps have been defined:

i. Calculation of the individual P-removal optimization potential by operational methods (chemical precipitation and/or increase of biomass P-uptake) according to the existing treatment technology;
ii. Estimation of the plant-specific costs for improved P-removal by enhanced P-incorporation into a biomass and/or chemical precipitation;
iii. Quantification of P-emissions from small WWTP in the entire river Warnow catchment, based on authority’s and operators’ survey data;
iv. Evaluation of the ecological relevance of the WWTP effluent reduction for the receiving water body;
v. Prioritization of the plants applying multiple cost-efficiency criteria.

The challenge and the novelty of this study was on the one hand to generalize the diversity of process engineering at rural WWTP to provide cost-efficient solutions and on the other hand to apply the ambient water quality-based approach for water resources management.

## Materials and methods

### Estimation of P-emissions from rural WWTP, receiving water body state and riverine loads

For the quantification of P-emissions and assessing the above given P-removal ratios, a database from the environmental state authority was used for the period of 2012–2015 for the 70 WWTP’s in the Warnow catchment. The annual means of the following data were used: effluent flow rates (m^3^ s^−1^), inflow and effluent P-concentration (mg L^−1^), and Chemical Oxygen Demand (COD, mgO_2_ L^−1^). Additional plant-specific technical data like activated sludge concentration (TS_ast_, g TSS L^−1^) and volume of the activated sludge tank (V, m^3^) have been requested and obtained in cooperation with WWTP operators.

To estimate the state of receiving water, we compared the water quality monitoring data (mean annual P-concentration) from state environmental agency with the target values according to the LAWA-RAKON criteria (LAWA-AO-RAKON 2005; Hering et al. 2010). Since in-stream transformation and transport processes (e.g. sediment dynamics, biomass uptake) and the impact of other sources (e.g. drains, agricultural point sources, groundwater) change the measurable P-load within short distances, representative monitoring points should be located only few kilometres downstream of discharge points with no relevant sources of sinks (e.g. lakes) in between. GIS-processing of the monitoring point and WWTP’s locations defining a tolerable distance of 5 km and neither intermediate side inflows nor standing waters identified only 19 measurement points to be “representative”. This scarce or at least highly uncertain data background is not sufficient to estimate the individual contribution of WWTP to the measured riverine loads in the tributaries and the entire Warnow catchment.

To overcome this, the water quality in the receiver is assumed to be ambient and P-concentration in the riverine water before WWTP-discharge was assumed to be equal to a geogenic background, where average value for the Warnow catchment is defined to be 0.05 mg L^−1^ (LAWA-AO-RAKON 2005). For the riverine background loads calculation, regionalised run-off rates, provided by the federal environmental agency, have been applied. The load after the WWTP-discharge is assumed to be composed only of background and WWTP-load (Eq. 1). Other P-emission sources like input from arable land, fertilizer application, urban surface water run-off or livestock slurry are intentionally not considered. Consequently, P-concentration (c_Model_) in receiving water body is simply modelled by mixing background load and WWTP-emission. Although this approach is obviously false, it is very helpful as shown below.

1 $$ {\text{C}}_{\text{Model}} = \frac{{0.05 \cdot {\text{Q}}_{\text{WB}} + {\text{c}}_{\text{P,effluent}} \cdot {\text{Q}}_{\text{effluent}} }}{{{\text{Q}}_{\text{WB}} + {\text{Q}}_{\text{effluent}} }} $$

### P-removal optimization potential of WWTP

#### Biological P-removal in ASS, SBR and OD plants

**Fig. 1:**
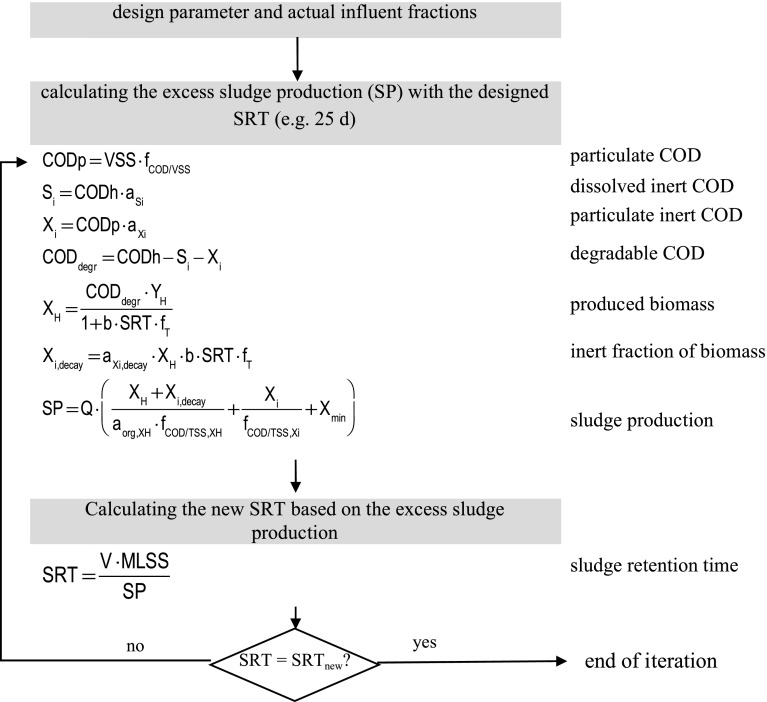
Iteration process for calculating the SRT by applying the COD approach

In activated sludge systems (ASS, including SBR and oxidation ditches(OD)), the removal of P is based on its incorporation into biomass, namely of heterotrophic bacteria. The P-incorporation into biomass is a function of COD in raw water (CODh) and sludge retention time (SRT). Reducing SRT by lowering the activated sludge concentration (expressed as TSS) and/or reducing the activated sludge tank volume can already enhance the P-removal. Where possible, installation of enhanced biological P-removal additionally improves the P-elimination. To calculate the plant-specific optimization potential we modified the German design guideline for activated sludge systems (DWA-A 131 2016) by replacing a constant factor for biological P-removal with a functional relationship between SRT and P-removal. The calculation schema and the model parameter are compiled in Fig. 1 and Table 2. For further details for calculating P-incorporation into biomass see supplementary materials.

**Table 2:** Parameter estimation for activated sludge systems in urban wastewater

Parameter	Symbol	Value	Literature
Yield	Y_H_	0.67 g_COD_ g_COD, deg_^−1^	DWA-A 131 (2016)
Decay rate	b	0.17 d^−1^	DWA-A 131 (2016)
Design temperature	T	10 °C	
Inert dissolved COD-fraction	a_Si_	5 % of COD_h_	DWA-A 131 (2016)
Inert particulate COD-fraction	a_Xi_	25 % of COD_p_	DWA-A 131 (2016)
Organic matter of biomass	a_org,XH_	92 %	Ramdani et al. (2010)
COD to VSS ratio	f_COD/VSS,XH_	1.45 g_COD_ g_VSS_^−1^	DWA-A 131 (2016)
Particulate COD-fraction	f_COD/VSS,Xi_	1.6 g_COD_ g_VSS_^−1^	DWA-A 131 (2016)
Chemical oxygen demand	COD	120 g_COD_ PE^−1^d^−1^	DWA-A 131 (2016)
Mixed liquor suspended solid	MLSS	50 g PE^−1^d^−1^	Friedrich (2014)
Mineral dry matter content	X_min_	20 g PE^−1^d^−1^	Imhoff (2007)
Temperature factor	f_T_	1.072^T−15^	DWA-A 131 (2016)
Organic biomass fraction	a_org, XH_	92 %	DWA-A 131 (2016)
Inert fraction of biomass	a_Xi, decay_	20 %	DWA-A 131 (2016)

In most cases, integration of EBPR requires only minor modifications on existing WWTP configuration and operational practice. For this, a sequence of anaerobic and aerobic phases is required, whereby the time interval of the anaerobic phase should be in the range of 1–2 h (Wentzel et al. 2008). Excess sludge has to be removed at the end of the aerobic phase, i.e. at the stage with maximum P-uptake. Detailed information on basics and application of EBPR are given in (Haandel and Lubbe 2012).

#### Biologic P-removal in biofilm systems and near natural systems

In contrast to ASS, P-removal in biofilm and near natural systems are much less predictable and largely governed by specific operational conditions. For biofilm systems (BS) with a separate storage for the excess sludge and primary sludge, the developed model is not suitable and cannot be satisfactorily, mathematically estimated. For those systems, a reliable and robust mathematical method to assess the optimization potential of biologic P-removal demands further investigations. Hence, biologic optimization measures for biofilm systems have not been taken into account. Besides, in the Warnow catchment a large number of sewage ponds are used. In these systems, P-removal can hardly be controlled in the treatment process. Therefore, the chemical precipitation is considered as a single optimization option for these facilities.

#### Chemical P-removal

Chemical precipitant (CP) is a universal approach for P-removal which can be applied additionally to each treatment technology. The process is based on precipitation with trivalent metals. The demand of precipitants and the related additional sludge production has been calculated (see supplementary materials). (Table 2).

### WWTP prioritization procedure

To prioritize the plants for introduction of P-removal, two criteria were defined. The criteria are constructed in a way that the final prioritization enables to identify the WWTP, where the P-removal will induce a cost-efficient and significant improvement of water quality in the receiving water body on different scales. They combine total emission, impact on ambient water quality and cost considerations. The criteria are defined as follows:

1. describes the maximum impact of the WWTP to the ambient water or achievable mitigation potential for the receiving water body;
2. is the cost efficiency to reduce total P-emission.

K1 is calculated according Eq. 2. It describes the maximum possible impact of the WWTP on the water body by assuming a natural background load of Phosphorous in the water body and hypothetical total removal of P. Other pollution sources in the river basin are neglected. Both assumptions (total P-removal and no other sources) do not reflect reality. However, the advantage is a normalized evaluation from the perspective of a desired natural ambient water quality. The higher K1, the higher the impact of the WWTP. Hence, the facility with the highest value gets rank 1. The K1 criterion addresses directly the desired good ecologic status of the individual receiving water body. Since this goal is not negotiable, cost comparison between different WWTP would only be useful for WWTP discharging in the same tributary. For that reason, cost considerations are intentionally not included in K1. The criterion K1 is calculated like follows:

2 $$ {\text{K}}_{ 1} = \frac{{{\text{c}}_{\text{P,effluent}} \cdot {\text{Q}}_{\text{effluent}} }}{{{\text{c}}_{\text{P,effluent}} \cdot {\text{Q}}_{\text{effluent}} + 0.05 \cdot {\text{Q}}_{\text{WB}} + }} \cdot 100\%, $$

where c is the concentration and Q the flow of the effluent and water body, respectively. In contrast, **K2**, addresses the total emissions in the Warnow catchment and seeks WWTP where these can be reduced with the best cost efficiency. The annual costs are calculated by a presumed zero discharge of P. Consequently, first the maximum biological P-removal is assessed and the still remaining P-concentration in the effluent is then considered to be removed by precipitation. So, the values in this criterion represent a mix of per-capita costs for biological P-removal enhancement, where it is possible, and chemical precipitation for all plants (K2, Eq. 3). The criterion K2 includes the sludge disposal costs (SC) of the additional sludge production due to biomass incorporation (f_S,EBPR_) and chemical precipitation (f_S,CP_), the precipitants costs (PC), the capital costs (CC) due to investments for the precipitation installations. According to operators experience in Germany, the investment costs for chemical precipitation differ between 2000 euros for the simple system to 20 000 euros for container solutions. For logistic reasons (interval of refilling the vessel) the simple solution can be used roughly up to a plant size of 800 PE (Tränckner et al. 2016). The annual capital costs (depreciation and interests) are calculated according to the German Working Group on Water Issues of the Federal States and the Federal Government (LAWA 2005). The depreciation period is uniformly defined with 15 years and the internal interest rate is assumed with 3.5 %. The costs for the precipitants differ widely depending on the respective product, the demand and logistic conditions. They also vary in time according to the market situation. In this case, the costs for precipitants are estimated with 0.80 euros/L_CP_. All cost calculation parameters are listed in Table 3.

3 $$ {\text{K}}_{2} = \frac{{{\text{SC}} + {\text{PC}} + {\text{CC}}}}{{{\text{X}}_{\text{a,P}} }}\left[ {\frac{{\sf C}\!\!\!\!\raise.8pt\hbox{=} }{{{\text{kg}}_{\text{P}} }}} \right] $$

4 $$  {\text{SC}} = \left( {{\text{c}}_{{{\text{P,}}\;{\text{bio  -  opt,}}\;{\text{effluent}}}} \cdot{\text{Q}}_{{{\text{effluent}}}} \cdot{\text{f}}_{{{\text{S,EBPR}}}}  + \left( {{\text{c}}_{{{\text{P,}}\;{\text{effluent}}}} {\text{ }} - {\text{c}}_{{{\text{P,}}\;{\text{bio  -  opt,}}\;{\text{effluent}}}} } \right)\cdot{\text{Q}}_{{{\text{effluent}}}} \cdot{\text{f}}_{{{\text{S,}}\;{\text{CP}}}} } \right)\cdot{\text{SD}} $$

5 $$ {\text{PC = Q}}_{\text{CP}} \cdot {\text{P}} $$

6 $$ {\text{CC}} = {\text{IC}} \cdot \frac{{{\text{i}} \cdot (1 + {\text{i}})^{\text{n}} }}{{(1 + {\text{i}})^{\text{n}} - 1}} $$

**Table 3:** Parameter estimation for activated sludge systems in urban wastewater

Calculation Parameters			References
PE	COD	120 g/d	DWA-A 131 (2016)
	L_P_	1.8 g/d	DWA-A 131 (2016)
	TSS	50 g/d	DWA-A 131 (2016)
Invest for precipitations station	IC	2000 euros	Market price
< 800 PE (compact station)	IC_1_	20 000 euros	Market price
800 PE–10 000 PE (container solution)	IC_2_		
Economic lifetime	n	15 a	assumption
Interest rate	i	3.5 %	Market price
Precipitation costs (40 % FeCl_3_)	P	0.80 euros/L	Market price
Sludge disposal costs	SD	300 euros/t TSS	Market price
Energy costs		0.25 euros/kWh	Market price
Stoichiometric factor for CP	f_S,CP_	6.8	Calculation
Biological P-incorporation factor	f_S,EBPR_	3 kg_sludge_/kg_P_	DWA-A 131 (2016)

## Results

**Fig. 2:**
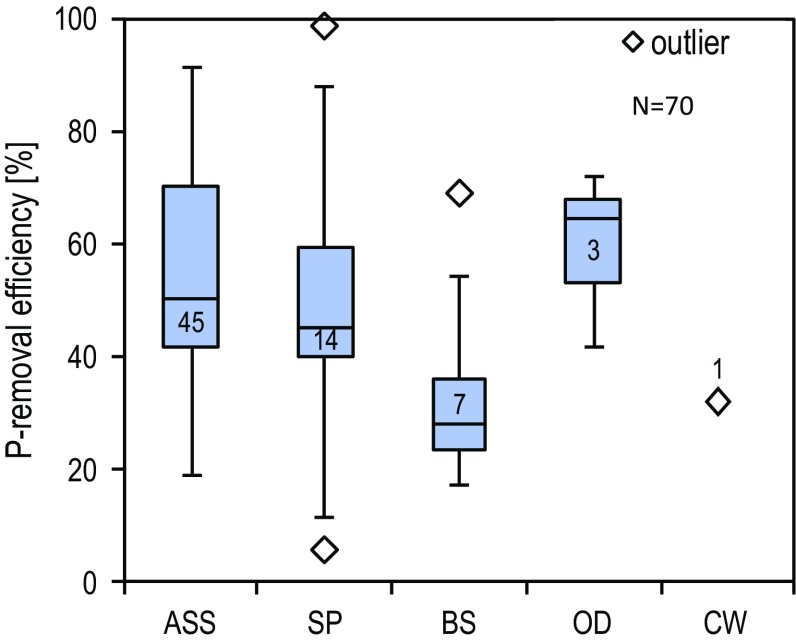
P-removal efficiency of small WWTP without P-precipitation according to treatment technology in the Warnow catchment. Comment: ASS is for Activated Sludge System, *SP* sewage pond, *BS* biofilm system, *OD* oxidation ditch, *CW* constructed wetlands

Measured effluent data of all WWTP in the class size 1–3 (excluding plants with P-precipitation) in Mecklenburg-Vorpommern reveal that P-removal strongly depends on the treatment technology. But for the same technology, significant plant-specific differences are observed, indicating an individual optimization potential of P-removal. Generally, the activated sludge systems (ASS) possess higher removal rates compared to other approaches. Technologically, this can be explained by the higher specific sludge production, and the higher P-incorporation compared, for example, to sewage ponds (SP) and biofilm systems (BS). Similar conditions are observed also for the entity of WWTP in the Warnow catchment (Fig. 2).

### Riverine water quality and loads estimation

**Fig. 3:**
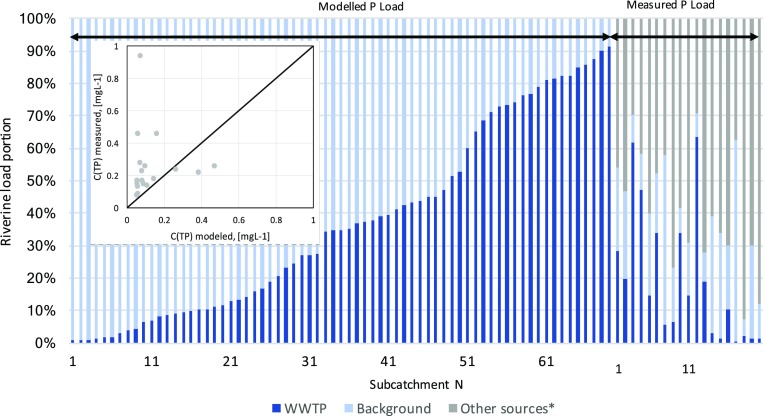
Validation of TP-concentrations in the receiving water body and TP-sources partitioning in relevant sub-catchments. “Other sources*” are identified only for catchments, where representative monitoring data were available

Comparison of modelled and available measured P-concentrations in WB shows, as expected, clear differences. Mostly, the P-concentrations are underestimated by the model (Fig. 3), since other anthropogenic P-sources are neglected. Reversely, the difference between the modelled concentration and the measured one can be regarded as miscellaneous P-emissions. In some cases, the measured concentrations are lower than modelled mixing concentration of natural background and WWTP effluent. This discrepancy might be explained either by outstanding retention capacity of the WB (river section) or by data inconsistency for the relevant measurement point (averaged grab samples, uncertain flow data).

In 60 % of all cases, the calculated riverine endogenous loads exceed the WWTP-emissions at the emission point by 150 % and more. But 26 % of the plants in the Warnow catchment emit more P than the assumed geogenic load of the upstream catchment (e.g. Figure 3, right).

### ASS model validation and P-removal potential

In the Warnow catchment, currently 58.1 % of total incoming P-load is removed by the small WWTP with less than 10^4^ PE. By introducing EBPR and reducing SRT, the percentage may be increased to 63.5 %. The remaining 36.5 % could be reduced by the application of the chemical precipitation (or other structural measures which have not been considered here). The limited effect is due to the already existing biological P-removal in many plants and the contribution of other systems which cannot be operationally improved easily. This applies in particular for pond systems. At least for the large ones (> 1000 PE) a change of technology should be considered, also because of the limited nitrification in the cold season.

**Fig. 4:**
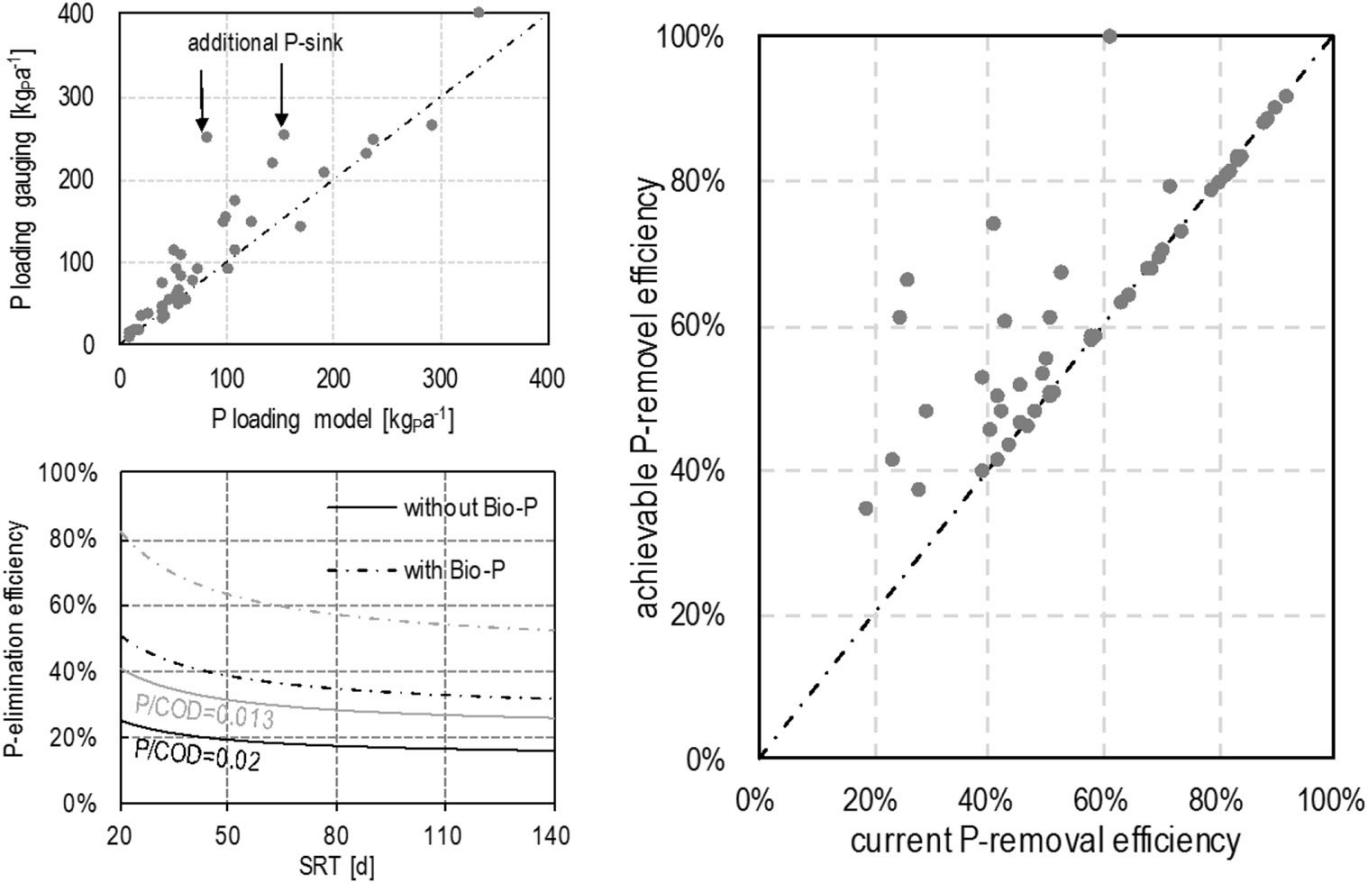
Validation of developed model by comparing the measured with the modelled P-removal (left, up); biological P-removal efficiency as a function of SRT with and without EBPR (left, down) and achievable P-removal efficiency of ASS in the Warnow catchment (right)

To evaluate the P-removal potential, the developed model for ASS has been validated and applied on 48 treatment plants, using ASS, SBR, and OD technologies, where required operational data were available. The developed model shows a good fit to operational data (Fig. 4, upper left). The spikes are explained by specific operational conditions representing an additional P-sink, which were tertiary treatment ponds in identified cases. Better model performance could be achieved by considering verified site-specific features of the treatment plants.

The model performance is presented in Fig. 4 (lower, left) for two treatment plants with different inflow quality (P/COD ratio). The markers in the lower lines give the actual and predicted removal efficiency. Moving along the relevant P/COD line left gives the achievable increase of removal by reducing SRT. This effect is rather limited. By additionally introducing EBPR, the dashed lines are valid. So, the markers on the dashed lines at SRT 25 days give the plant-specific maximum removal. (Figure 4, lower, left).

As depicted in Fig. 4 (right), 26 out of 48 ASS facilities are located on the line. Hence, their current biological P-removal efficiency has already achieved the optimum, i.e. these plants apply EBPR at a technologically reasonable SRT. For the remaining 22, a potential P-removal improvement with operational methods of 5–42 % with the mean value of 14% have been identified. Further P-removal increase is possible by chemical precipitation.

### Prioritization of WWTP in the Warnow catchment

**Fig. 5:**
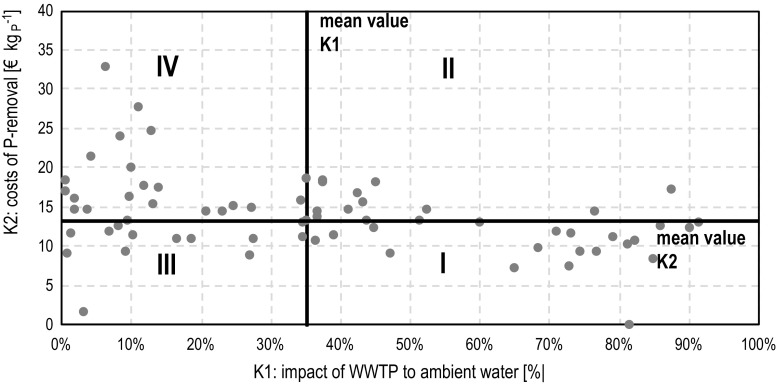
Scoring of the rural WWTP’s in Warnow catchment with the criteria K1 and K2

For the calculation of K1 the target P-concentration in the effluent was considered to be zero. In the case of hypothetical complete P-removal in rural WWTP a maximum possible reduction of the riverine load is calculated, varying from 0.7 to 91.5 % in relevant water bodies under assumed ambient water quality conditions (Fig. 5). These criteria value evaluates the treatment plant capacity in relation to the receiving WB capacity, i.e. the larger plants discharging into smaller water bodies the higher the relevance.

Analogous to K1 criterion, a zero discharge was assumed in K2 and is achieved by first using the available biological reduction potential, followed by precipitation for the remaining load. In this criterion, the costs for reducing P are evaluated. Facilities with low costs for the measures should be prioritized at first.

In Fig. 5, both criteria are arranged for all WWTP in an XY-diagram. To elucidate the results, it is divided into four sections taking the median value of the respective criterion as dividing line. According to criterion K1, plants on the right have a high eutrophication impact, hence, facilities in sector I kg_P_ and II are of interest when P at the local receiving water body must be reduced. Criterion K2 gives the costs per reduced P-emission. Plants with low K2 values have a good cost efficiency and should be prioritized when reduction of total emissions (e.g. in this case in the Baltic) is intended. For example, plants in sector IV show a low eutrophication impact and high costs. Therefore, these plants should be optimized at last. Weighting both parameters require a definition of strategic goals and/or a further multi-criterial decision making. A very simple mathematic key figure could be derived by dividing the eutrophication impact (K1) with the costs of P reduction (K2). The higher this value, the higher the impact and lower the costs. Thus, plants with the highest quotient K1/K2 should be prioritized. But, since K1 (due to the widely differing flows of the receiving water bodies) is varying much stronger than K2, this puts a strong weight on K1. Separate ranking and subsequent prioritization would ensure more equality between both parameters.

**Fig. 6:**
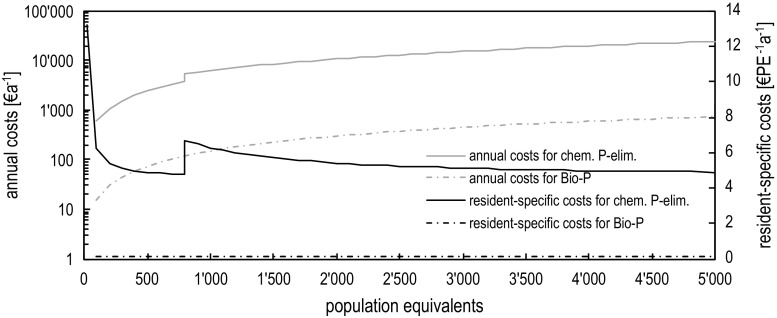
Comparison of the annual and resident-specific costs of biological and chemical methods. Conditions: disposal costs are 300 euros t^−1^ excess sludge

Figure 6 shows the calculated total annual and the per-capita costs for both optimization options. While the per-capita costs are nearly negligible for the EBPR, the chemical precipitation costs range between 5 and 6.5 euros CAP^−1^a^−1^. The jump at 800 PE is due to the defined technology change from the compact dosage station to the container solution to guarantee a minimum two-week interval of refilling the precipitants. Depending on specific inflow P-concentration the point of technology switch is plant specific, but the tendency remains. The significant cost differences between both options reveal that the operational improvement potential should be carefully checked before asking for chemical precipitation in small WWTP. Unfortunately, EBPR and other improvement measures are less reliable over time and will therefore not always meet fixed discharge limits. But since Phosphorous is a non-toxic eutrophication parameter, agreeing less strict target limits with an annual mean value for P-discharge could be a reasonable compromise between ecologic and economic considerations.

## Discussion

Although the total P-emissions from rural WWTP are high in the Warnow catchment the local eutrophication potential at the discharge point is only relevant for one-third of the cases in comparison to the endogenous input. Obviously, by considering other P-emission sources the portion of the WWTP-emissions in the riverine load will decline. The relevance of the TP input from rural WWTP in Warnow catchment in comparison to the other P-sources cannot be soundly evaluated based on the method presented here. This would require a comprehensive P-cycle model for the catchment. To improve the significance of the results, ambient state of water quality in this study might be replaced by measured values or derived from a validated TP balance model of the catchment and the relevant sub-catchments.

Calculating biological P-removal in ASS as a function of sludge retention time is a significant extension for the German design standard DWA-A 131. Especially in rural areas WWTP are often heavily underloaded due to a significant decrease of population. As shown, a reasonable adaption of operational conditions to the changed WWTP-load can significantly improve P-removal. A reasonable decrease of the SRT and simultaneous introduction of EBPR to WWTP in Warnow catchment will enable to remove additional 20 % of the emissions into aquatic environment.

The biological P-removal in temperate climate is known to be season dependent, thus to achieve fixed discharge limits over an entire year an additional chemical precipitation facility would be mandatory. Moreover, many ASS plants are already operating on the highest possible removal rate. For those plants, further improvements would always require chemical precipitation.

Chemical precipitation is mostly applicable respecting some boundary conditions, but it is quite expensive, especially for plants with few PE. To promote the cost-efficient improvement of biological P-removal, strict effluent standards could be replaced by more flexible target values. Customized process engineering solutions are more challenging but can significantly save costs. One example, is the consequent separate storage of excess primary and secondary sludge to avoid unintended P release. This applies in particular for trickling filters which generally produce both sludge types.

The prioritization criteria composed in this study combine local impact on ambient water, total emission and costs, and enable for a target-oriented prioritization. Certainly, more reliable boundary conditions, like a higher temporal and spatial resolution of quality and flow data, more case specific cost estimations or consideration of seasonal variations of biological P-removal would increase the significance of the selection within the criteria. But in this case the scale of the study should be reduced.

The study shows that the point-oriented measures can be outlined for matter source control in the catchment even with limited data availability. The prioritization criteria might be weighted according to the interest of the decision makers. This can have an advantage for the catchments with higher diversity of treatment technologies on the WWTP, strongly impacted/protected water resources, or other objectives of the prioritization.

## Conclusions

Accumulated for a river basin, small WWTP can have higher P-emissions than large plants. But their environmental relevance on local scale is very case specific. Therefore, the requirements or measures to reduce emissions should be justified by necessity and possibilities for the specific plant and receiving water body.

ASS systems should first use the operational potential of P-removal by reducing SRT to reasonable values and if possible by introducing EBPR. These measures are for the plant very cost effective. But the technological options must be concerned not only with regard to the local treatment technology but also to the logistics of sludge treatment.

Conventional chemical precipitation might be applied to improve P-removal for the entire variety of treatment technologies on rural WWTP’s. The related costs between 5 and 6.5 euros Cap^−1^a^−1^ are affordable but must be well reasoned (see Fig. 6).

The developed prioritization algorithm represents an approach for the sustainable water resources management, where the interests of several stakeholders are considered, like WWTP operators, local communities and environmental authorities. Due to the low data demand and the mathematic simplicity, its practical implication is expected with high acceptance degree.

## Electronic supplementary material

Below is the link to the electronic supplementary material.

Supplementary material 1 (PDF 603 kb)
