# Supplementary material for: Multi-criterial evaluation of P-removal optimization in rural wastewater treatment plants for a sub-catchment of the Baltic Sea
Source: Ambio. 2017 Nov 21;47(Suppl 1):93–102. doi: 10.1007/s13280-017-0977-8 (PMC5722745; doi:10.1007/s13280-017-0977-8)
Supplement: Supplementary file 1 — Supplementary material 1 (PDF 603 kb) [file 13280_2017_977_MOESM1_ESM.pdf]

Ambio

Electronic Supplementary Material

This supplementary has not been peer reviewed.

Title: **Multi-criterial evaluation of P-removal optimization in rural wastewater treatment plants for a sub-catchment of the Baltic Sea**

Authors: Michael Cramer, Tatyana Koegst, Jens Traenckner

Calculating P incorporation into biomass In Germany, design of ASS is based on a COD balance (DWA-A 131 2016). Thereby, P incorporation into biomass is determined via a constant relationship between COD and P removed, without taking the SRT into account. In this work, the calculation of P incorporation is modified by linking the P removal to the biomass production as function of SRT. Since biomass production is a function of SRT and SRT depends on sludge production, for a given plant, an iterative procedure, starting with an estimate of SRT, is required. Total sludge production (SP) is calculated according to the scheme in Fig. 1. The sludge production is determined by inert (X_i) and mineral (X_{min}) compartments of particulate COD (CODp) as well as by derivatives of degradable COD: biomass produced (X_H) and biomass reduced by decay ($X_{i,}$ decay). Since the COD balance yields only the organic part of biomass, which is about 92% (Ramdani et al. 2010), calculated total biomass is increased by this ratio. The CODp is estimated from the inflow volatile suspended solids (VSS). If this value is unknown, it can be estimated as a difference between total suspended solids (TSS) and an estimate for the mineral fraction. Suggested coefficient values for COD fractioning, decay rate and transferring COD into TSS are given in Table 2.

The degradable COD, which is the CODh without inert particulate (X_i) and inert dissolved compartments (S_i), is used to calculate biomass production (X_H), considering a decay rate (b). The dissolved inert fraction (S_i) of COD_h not contributing to biomass production, is estimated with 5% of the CODh (Haandel and Lubbe 2012). A good guess is also the difference between effluent COD and effluent COD of total suspended solids (TSS). The decay products are not completely utilized for new biomass production. The remaining inert fraction of biomass ($a_{Xi,decay}$) contributes to the total sludge production (SP). With stoichiometric factors, the different COD fractions can be transferred into TSS.

For calculating P removal, only the produced biomass X_H is relevant. About 2.5% of total P in biomass is incorporated into ordinary heterotrophic organisms (Haandel and Lubbe 2012). This value can increase up to 5–8% by introduction of EBPR (Haandel and Lubbe 2012). Depending on actual conditions, biological P incorporation is calculated here with Eq. 10, where $f_{P,BM}$ equals 0.025 for plants without or 0.05 for plant with enhanced biological removal.

$$X_{P,removed} = \frac{X_H}{f_{COD7TSS,XH}} \cdot f_{P,BM} \quad (1)$$

To enhance biomass production, the SRT should be shortened either by reducing TSS in the reactor and/or by reducing reactor volume. The latter approach can be easily applied in SBR systems by controlling the filling level. If sludge is directly valorized in agriculture, a minimal SRT of 25 days is required as sludge stabilization criterion. If it is anaerobically digested or incinerated, the SRT can be further decreased down to values still assuring proper nitrification. At very low TSS concentrations, floc formation can be disturbed. To avoid poor sludge settling properties, a minimum TSS concentration of 1.5 gL^{-1} was defined (eq. 11). A remaining operational question is the tolerable lowering of the water level in SBR systems. The assumption in this approach is that the sludge level declines similarly to the TSS reduction in aqueous phase, hence, the water level can be reduced with the same factor. The limiting value for lowering the water level is the location of the sludge blanket. In most cases a reduction of the water level by 50% (along with an according TSS reduction) should be operational feasible.

This means, that the minimal possible sludge age (SRT_{min}) for SBR plants can be calculated as follows:

$$SRT^{*}_{min} = \frac{SRT}{\left(\frac{TSS}{1.5}\right)^{2}} \tag{2}$$

Calculating chemical P removal The required P removal load $X_{P,CP}$ is set to current effluent load to assume a zero discharge:

$$X_{P,CP} = X_{P,effluent} \tag{3}$$

The demand of chemical precipitants Q_{CP} for a target value c_{target} is calculated by:

$$Q_{CP} = \frac{M_{CP}}{M_{P}} \beta \cdot (c_{P,effluent} - c_{target}) \cdot Q_{effluent} \cdot \frac{M_{CP} + \sum_{i=1}^{n} x_i \cdot M_{AS,i}}{M_{CP} \cdot c_{solution} \cdot \rho_{solution}}$$

where M is the molar mass of CP, P and attendant solution (AS), respectively, β is the stoichiometric excess factor, Q the volume, c the concentration and ρ is the density of the precipitants solution. X_i is for stoichiometric factor.

Chemical precipitation of P increases the mineral sludge production. The additional sludge production due to chemical precipitation SP_{CP} is calculated by:

$$SP_{CP} = \left(c_{P,effluent} - c_{P,bio-opt,effluent}\right) Q_{effluent} \cdot f_{S,CP}$$

where $f_{S,CP}$ is the stoichiometric factor for CP (see Table 3).
